# Association of body mass index with bladder cancer risk: a dose-response meta-analysis of prospective cohort studies

**DOI:** 10.18632/oncotarget.16722

**Published:** 2017-03-30

**Authors:** Limin Zhao, Xiaoqin Tian, Xueyan Duan, Yongxiu Ye, Min Sun, Junfang Huang

**Affiliations:** ^1^ Department of General Medicine, Shenzhen Longhua New District Central Hospital, Shenzhen, Guangdong, P.R. China; ^2^ Department of Endocrinology, Shandong Liaocheng City Central Hospital, Liaocheng, Shandong, P.R. China; ^3^ Department of Urolgoy, The Xinjiang Uygur Autonomous Region Peoples Hospital, Uygur, Xinjiang, P.R. China

**Keywords:** body mass index, bladder cancer, risk, dose-response, meta-analysis

## Abstract

Prospective epidemiologic studies on the association between body mass index (BMI) and bladder cancer yielded inconsistent findings. This study sought to quantitatively summarize the evidence by performing a dose-response meta-analysis on prospective cohort studies. Eligible studies were retrieved via PubMed and Embase databases, and by manual review of the references. Linear and nonlinear trend analyses were conducted to explore the relationships between BMI and bladder cancer risk. Meta-analyses on the categories of overweight and obesity were also conducted. The summary relative risk (SRR) was estimated. Heterogeneity across the studies was explored through subgroup analyses based on gender, age, year of publication, sample size, assessment of BMI, geographic location, physical activity and family history of cancer. A total of 14 prospective cohort studies involving 12,642 cases were included. Result of the dose-response analysis showed a nonlinear positive relationship between BMI and bladder cancer (SRR = 1.03, 95% CI: 1.01-1.06, *P*-nonlinearity =0.031), suggesting that per 5 kg/m^2^ increment on BMI corresponded to a 3.1 % increase of bladder cancer risk, especially BMI exceed 30kg/m^2^. Furthermore, significant positive association was also observed between obesity category and bladder cancer risk (SRR: 1.10, 95%CI: 1.03-1.17). In summary, this dose-response meta-analysis suggests a nonlinear positive association between BMI and bladder cancer risk. Further studies are required to confirm these findings and elucidate the pathogenic mechanisms.

## INTRODUCTION

Bladder cancer is among the most common malignancies of the genitourinary tract all over the world [1]. Approximately 75% of new diagnosed cases are non-invasive disease, but they have a high rate of recurrence and progression despite treatment by transurethral resection combined with intravesical chemotherapy. The remaining ~25% of cases are muscle invasion. They still have poor outcome despite systemic therapy concerning radical surgery or radiotherapy [2]. Because the rising incidence of bladder cancer is alarming, a substantial challenge has been presented to public health. It is an increasing common view that making effective control measures to prevent form bladder cancer is more effective and significant [3]. However, currently, there are limited effective preventive measures against it. Many studies have focused on risk factors for bladder cancer. It has been well established that cigarette smoking, occupational exposure to aromatic amines, and chronic schistosoma infection are significant etiological factor for bladder cancer [4, 5].

Excess bodyweight, whether in people who are overweight or obese, is increasingly recognized as an important risk factor for many common cancers [6]. Body mass index (BMI), defined as body weight in kilograms divided by the square of height in meters, is used as one of the most commonly anthropometric measure [7]. The association between BMI and bladder cancer risk has received much attention in the past few years, but the findings were still controversy [8–21]. To date, there has not any study that examines the exact shape of the dose-response relationship between BMI and bladder cancer risk based on prospective cohort studies.

Thus, we conducted this meta-analysis to quantitatively summarize the evidence on the association of BMI with bladder cancer risk by performing a dose-response meta-analysis on the basis of prospective cohort studies.

## RESULTS

### Study selection and characteristics

**Figure 1:**
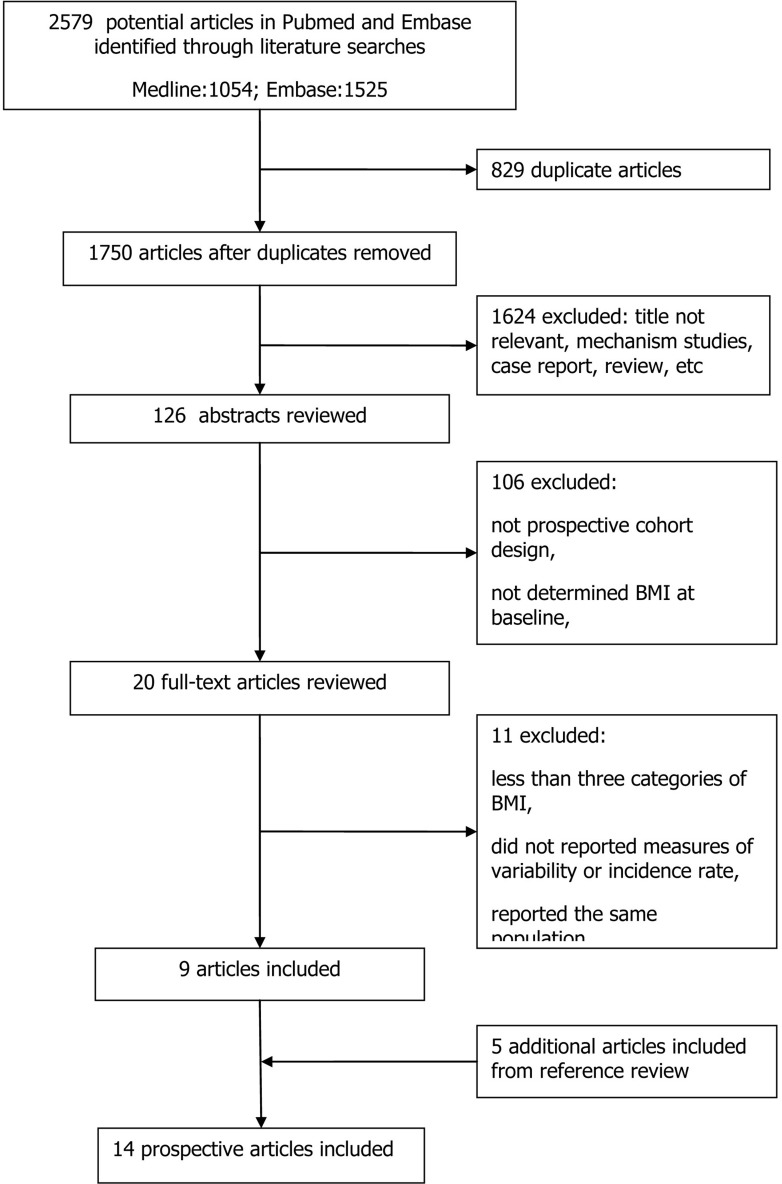
Flow chart of the study selection

Figure 1 shows a flow chart of our selection process. Our initial search yielded 2579 independent citations *via* database search. Of these, 2453 articles were excluded considering of potential value after excluding the duplicate or not relevant articles, mechanism studies, case reports or reviews. Furthermore, 106 articles were excluded following an initial screen of abstracts, and the other 11 papers were excluded after reviewing full-texts because of a lack of results reported BMI and bladder cancer. The remaining 9 articles finally met all the selection criteria. Of note, 5 additional articles were included from references review. Thus, a total of 14 articles (involving 12,642 cases) with a sample size of 5,640,760 participants were included in our meta-analysis.

The main characteristics of the included studies are shown in Table 1. Among the included studies, BMI was either self-reported, or measured by investigators. The study duration, sample size, and potential confounders adjusted for varied substantially among individual studies. All studies were published between 2002 and 2014, with the mean duration of follow-up varying from 4.28 to 19 years. The sample size ranged from 37,459 to 1,222,630. Of the fourteen studies, four were conducted in USA [13, 15, 16, 21], three in Sweden [17–19], two in Korea [12, 20], two in international multicenters [8, 10], and one in the United Kingdoms [14], Austria [19] and China each [9]. Few studies controlled for physical activity and family history of cancer, however, all the included studies controlled for cigarette smoking. All articles included were published in English, except for one in Chinese [9]. The Newcastle-Ottawa Scale (NOS) was applied to assess the quality of the included studies and the results showed all the studies were of high quality, with NOS score ≥7.

**Table 1:** Characteristics of prospective cohort studies of body mass index and risk of bladder cancer

Study	Area	Follow-up period (years)	Mean age (year-old)	Sample size	Cases	NOS	Cut-off	Outcome (RR 95%CI)	
								Male	Female
Roswall 2014 [8]	Denmark, Sweden, Germany, The Netherlands, United Kingdom, France, Italy, Spain, Greece	1992-2008 (11.7y)	52.3	390,878	1,391	9	Q1 Q2 Q3 Q4	Reference 1.20 (1.01-1.43) 1.11 (0.93-1.33) 1.25 (1.04-1.50)	Reference 1.07 (0.80-1.43) 0.97 (0.73-1.30) 0.91 (0.68-1.23)
Guo 2014 [9]	China	2006-2011 (4.28y)	51.1	106,630	64	7	<18.5 18.5-24.0 24.0-28.0 ≥28.0	1.24 (0.38-4.08) Reference 0.44 (0.23-0.84) 0.86 (0.40-1.87)	
Haggstrom 2011 [10]	Norway, Austria, Sweden	11.7y	44	578,699	1,914	9	Q1 Q2 Q3 Q4 Q5	Reference 1.06 (0.89-1.28) 1.06 (0.88-1.26) 0.97 (0.80-1.16) 1.13 (0.94-1.35)	Reference 1.00 (0.66-1.51) 1.00 (0.67-1.50) 0.67 (0.44-1.03) 0.87 (0.58-1.32)
Larsson 2008 [11]	Sweden	1998-2007 (9.3y)	NR	43,480	388	8	18.0-24.9 25.0-29.9 30.0-34.9 ≥35.0	Reference 0.98 (0.79-1.20) 0.92 (0.62-1.34) 0.79 (0.29-2.14)	
Jee 2008 [12]	Korea	1992-2006 (10.8y)	47.2	1,213,829	2,439	9	<20.0 20.0-22.9 23.0-24.9 25.0-29.9 ≥30.0	0.84 (0.68-1.04) 0.91 (0.78-1.05) Reference 1.19 (1.01-1.40) 1.02 (0.52-1.97)	0.57 (0.31-1.03) 0.74 (0.49-1.11) Reference 1.10 (0.75-1.62) 0.74 (0.27-2.06)
Koebnick 2008 [13]	USA	1995-2003 (8y)	61.2	471,760	1,719	8	18.5-24.9 25.0-29.9 30.0-34.9 ≥35.0	Reference 1.21 (1.07-1.37) 1.21 (1.03-1.43) 1.25 (0.96-1.63)	Reference 0.84 (0.62-1.15) 1.38 (0.96-1.96) 1.37 (0.87-2.18)
Reeves 2007 [14]	United Kingdom	1996-2001 (5.4y)	55.9	1,222,630	615	7	<22.5 22.5-24.9 25-27.4 27.5-29.9 ≥30		0.99 (0.83-1.19) Reference 1.14 (0.97-1.34) 1.15 (0.93-1.41) 1.07 (0.88-1.30)
Holick 2006 [15]	USA	1986-2002 (16y)	48.8	162,535	866	7	18.0-22.9 23.0-24.9 25.0-26.9 27.0-29.9 ≥30.0	Reference 1.11 (0.84-1.47) 1.14 (0.86-1.51) 1.12 (0.83-1.51) 1.01 (0.68-1.50)	Reference 1.04 (0.78-1.38) 1.23 (0.90-1.69) 1.09 (0.75-1.58) 1.31 (0.91-1.89)
Cantwell 2006 [16]	USA	1980-1998 (15.3y)	55.4	54,308	167	8	<18.5 18.5-25 25-30 30-35 >=35		0.55 (0.14-2.24) Reference 1.05 (0.73-1.50) 1.28 (0.73-2.25) 0.83 (0.26-2.63)
Samanic 2006 [17]	Sweden	1971-1999 (19y)	34.3	362,552	2,030	8	18.5-24.9 25.0-29.9 ≥30	Reference 0.94 (0.86-1.03) 0.91 (0.76-1.09)	
Lukanova 2006 [18]	Sweden	1985-2003 (8.2y)	46	68,786	98	9	18.5-24.9 25.0-29.9 ≥30.0	Reference 1.17 (0.71-1.94) 1.39 (0.64-2.79)	Reference 0.76 (0.26-2.02) 2.12 (0.77-5.43)
Rapp 2005 [19]	Austria	1985-2002 (9.9y)	42.2	145,931	229	9	18.5-24.9 25-29.9 30-34.9	Reference 0.81 (0.59-1.11) 0.74 (0.45-1.22)	Reference 1.35 (0.74-2.48) 1.60 (0.76-3.36)
Oh 2005 [20]	Korea	1992-2001 (10y)	40.1	781,283	610	8	<18.5 18.5-22.9 23.0-24.9 25.0-26.9 27.0-29.9 ≥30.0	1.76 (1.09-2.84) Reference 1.20 (0.99-1.45) 1.12 (0.89-1.41) 1.16 (0.83-1.61) 0.70 (0.22-2.19)	
Tripathi 2002 [21]	USA	1986-1998 (13y)	NR	37,459	112	7	≤22.89 22.90-25.02 25.04-27.43 27.46-30.67 ≥30.69		Reference 0.88 (0.52-1.48) 0.66 (0.38-1.16) 0.58 (0.32-1.04) 0.53 (0.29-0.96)

NOS: Newcastle-Ottawa Scale; Q1~Q5: quartiles of exposures; NR: No reported.

### Overall analyses

**Figure 2:**
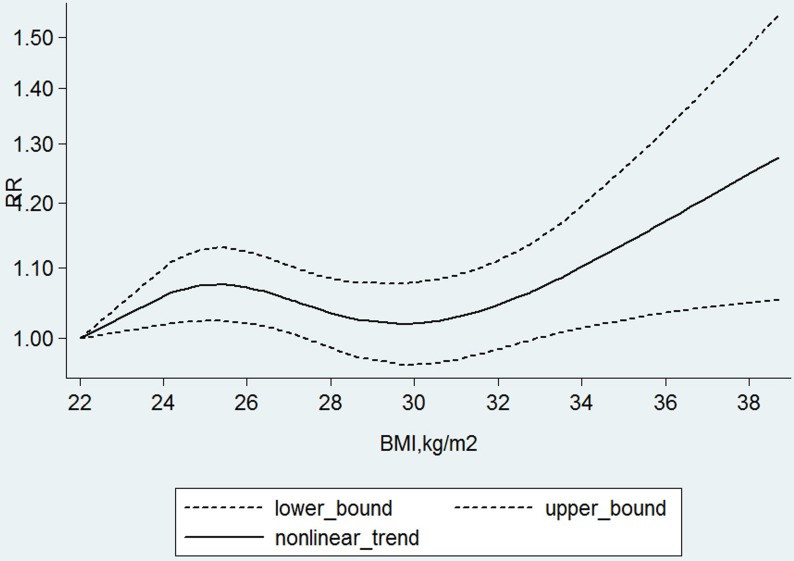
The nonlinear dose-response meta-analysis of body mass index (BMI) and risk of bladder cancer

When we fitted the linear dose-response model to regress the relative risks of bladder cancer on per 5 kg/m^2^ increase in BMI for each studies, the dose-response meta-analysis in random-effects model across all the studies showed that there was a weak positive association between BMI and bladder cancer risk, indicating that per 5 kg/ m^2^ increment on BMI corresponded to a 3.1 % increase of bladder cancer risk (The summary relative risk (SRR): 1.03, 95%CI: 1.01-1.06, *p* = 0.039) with evidence of no problems in the fitted model (Q = 51.85, *p* = 0.099). There was evidence of a potential nonlinear association between BMI and risk of bladder cancer(X^2^ = 6.95, *p* = 0.031), which showed that there seem to be a slight higher risk in the range of 24-28 kg/m^2^ and a further, with the curve becoming steeper and almost linear after the BMI exceed 30kg/m^2^ compared to the average the reference of 22kg/m^2^ (Figure 2).

**Figure 3:**
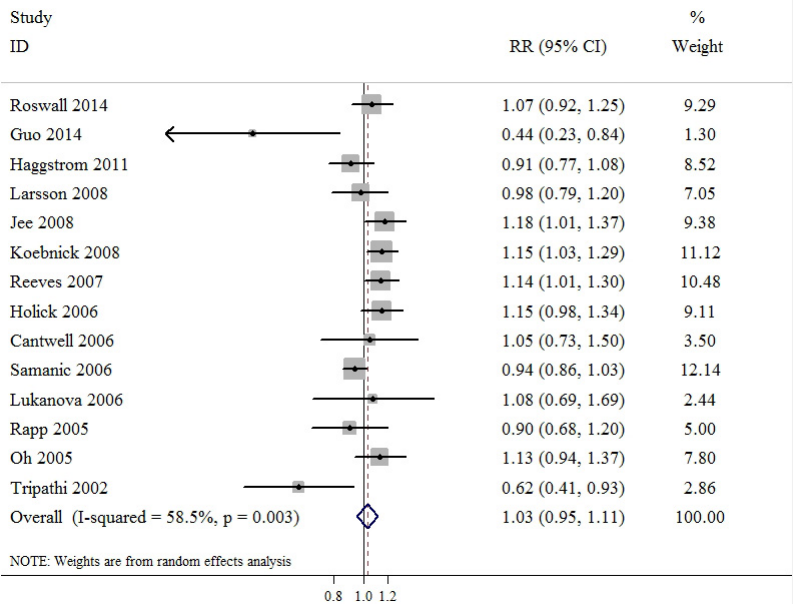
Meta-analysis of studies that examined the association between overweight category and bladder cancer risk

**Figure 4:**
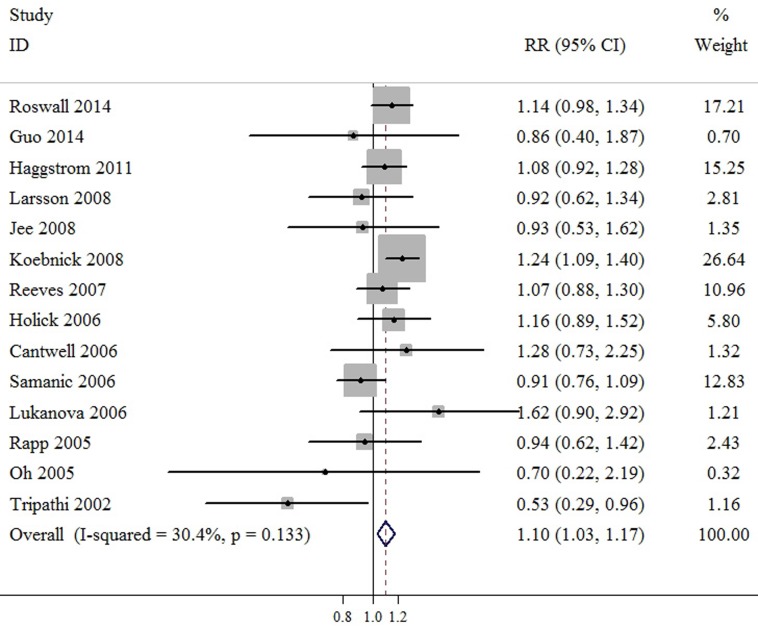
Meta-analysis of studies that examined the association between obesity category and bladder cancer risk

Compared to normal weight, meta-analyses based on BMI categories found that the SRR was 1.03 (95%CI: 0.95-1.11) for overweight, and 1.10 (95%CI: 1.03-1.17) for obesity. There was evidence of heterogeneity among studies for obesity category (*P* value for heterogeneity = 0.003, I^2^ = 58.5%), but not for overweight category (*P* value for heterogeneity = 0.133, I^2^ = 30.4%). These results indicated that overweight was not associated with bladder cancer risk, but obesity was associated with increased by 10.0% risk of bladder cancer (Figure 3 & Figure 4). Further sensitivity testing *via* the exclusion of a single study at a time suggested that no single study influenced the overall results in this meta-analysis. Furthermore, no statistical evidence of publication bias was found in this meta-analyses, as assessed by Begg’s and Egger’s tests for overweight (p-Begg = 0.584; p-Egger = 0.619), and obesity (p-Begg = 0.458; p-Egger = 0.579), respectively.

### Subgroup analyses

When studies were stratified by gender, age, year of publication, sample size, assessment of BMI, geographic location, physical activity and family history of cancer, the results in subgroup analyses were consistent with the primary findings. Begg and Egger tests provided no evidence of substantial publication bias in any subgroup (not shown). We performed sensitivity analyses and found that none of the studies considerably affected on the overall or stratified estimate between BMI and bladder cancer in this meta-analysis (data not shown). In addition, little variability was observed among the most subgroup analyses.

## DISCUSSION

It has been reported that BMI has been associated with worse outcomes in several solid malignancies. Weight gaining is known to predispose to a number of cancers in certain populations, including cancer of colon, kidney, breast, endometrium, gallbladder and so on [22]. However, evidence for bladder cancer is sparse. Furthermore, it did not examine the exact shape of the dose-response relationship between BMI and risk of bladder cancer. Compared to the previous quantitative review by Qin [23] and Sun et al [24]. With recently accumulating evidence, our meta-analysis was to explore the association between BMI and bladder cancer risk based on prospective cohort studies with huge sample size, and the dose-response association between BMI and bladder cancer risk was also examined.

**Table 2:** Summary of meta-analysis results for BMI and bladder cancer risk

Analysis specification	Studies	SRR (95%CI)	*I*^2^ (%)	P*
Overweight				
All	14	1.03 (0.95-1.11)	30.4	0.133
Mean age				
≥50	5	1.01 (0.43-1.21)	24.8	0.104
<50	7	0.94 (0.87-1.34)	0.0	0.419
Year of publication				
≥2008	6	1.03 (0.89-1.09)	3.4	0.411
<2008	8	1.05 (0.78-1.28)	0.0	0.633
Follow-up duration				
≥10 years	8	0.96 (0.57-1.23)	0.0	0.857
<10 years	6	1.07 (0.89-1.18)	16.2	0.153
Sample size				
≥1,000,000	4	1.05 (0.45-1.37)	0.0	0.477
<1,000,000	10	1.01 (0.96-1.10)	54.6	0.031
Assessment of BMI				
Measured	6	1.10 (0.91-1.23)	53.4	0.008
Self-reported	7	1.02 (1.86-1.15)	12.9	0.301
Geographic area				
Asia	3	1.02 (0.97-1.08)	0	0.675
Europe	4	1.06 (0.97-1.73)	47.0	0.100
America	4	1.03 (0.79-1.34)	0.0	0.861
Multi–international centers	2	1.09 (0.98-1.56)	12.9	0.351
Physical activity				
Yes	2	1.07 (0.94-1.29)	35.9	0.191
No	12	1.15 (1.00-1.98)	42.7	0.121
Family history of cancer				
Yes	2	1.15(0.93–1.29)	12.8	0.543
No	12	1.06(1.00–1.19)	36.1	0.087
Obesity				
All	14	1.10 (1.03-1.17)	58.5	0.003
Mean age				
≥50	6	1.19 (1.08-1.22)	19.9	0.267
<50	6	1.01 (1.09-1.68)	56.8	0.061
Year of publication				
≥2008	6	1.10 (1.53-1.89)	27.1	0.171
<2008	8	1.02 (1.14-1.40)	66.2	0.000
Follow-up duration				
≥10 years	8	1.30 (1.02-1.39)	0.0	0.424
<10 years	6	1.07 (1.04-1.74)	10.3	0.231
Sample size				
≥1,000,000	4	1.14 (1.01-1.61)	24.8	0.200
<1,000,000	10	1.04 (1.08-1.54)	37.1	0.112
Assessment of BMI				
Measured	7	1.09(1.02–1.13)	12.5	0.354
Self-reported	6	1.24(1.09–1.40)	32.4	0.187
Geographic area				
Europe	3	1.84 (1.55-2.19)	0.0	0.651
America	4	1.97 (1.40-2.75)	18.8	0.214
Asia	3	2.29 (1.46-3.58)	17.1	0.341
Smoking				
Yes	2	1.11(1.05–2.16)	24.1	0.145
No	12	1.17(1.05–1.46)	8.81	0.653
Family history of cancer				
Yes	2	1.13(1.07–1.19)	29.9	0.201
No	12	1.09(1.04–1.13)	1.83	0.412

BMI: Body mass index, SRR: The summary relative risk, *P value for heterogeneity.

In this updated meta-analysis, fourteen prospective cohort studies comprising 5,640,760 participants were included in the current meta-analysis to explore the dose-response relationship between BMI and bladder cancer risk. Overall, the dose-response meta-analysis in random-effects model across all the studies showed that there was a potential nonlinear association between BMI and bladder cancer risk and the risk increased by 3.1% for each 5 kg/m^2^ increase. Further our finds indicated that a positive association was found between bladder cancer and obesity compared with normal weight, with little variability. Considering the unsatisfactory exploring in single study and only a weak association between BMI and bladder cancer risk was detected based on linear trend analysis, we conducted a nonlinear trend analysis which also showed a significant fitting, and implying that further meta-analyses based on categories were necessary. The results of meta-analyses based on overweight and obesity confirmed our assumption that bladder cancer risk was increased in particular range of BMI. Notably, no significant association was observed between overweight category and bladder risk

A greater heterogeneity was detected in those studies on obesity category likely due to differences among study populations, model selection, analytic methodology and exposure assessment. Subgroup analyses were conducted to investigate the sources of the observed heterogeneity among studies. The results in subgroup analyses were consistent with the overall results. A significant proportion of the observed heterogeneity may be explained through the subgroup analyses based on gender, age, year of publication, sample size, assessment of BMI, geographic location, physical activity and family history of cancer. Little variability was observed through the stratified analyses. Furthermore, we have, however, shown an absence of publication bias in these meta-analyses with either the Egger’s or Begg’s tests. Sensitivity analyses showed none of the studies considerably affected the summary associations between BMI and risk of bladder cancer.

Several biologic mechanisms contribute to the association between excess weight and risk of cancers, including insulin resistance, resultant chronic hyperinsulinaemia, increased bioavailability of steroid hormones and localized inflammation [25]. The underlying mechanism involved in the association between BMI and bladder cancer risk is uncertain. One possible cause is the biologic mechanisms that link excess weight and bladder cancer risk. It is well-known that excess body fat is associated with the elevated production of insulin, which is a mitogenic stimulation factor that may enhance tumor growth by increasing free insulin-like growth factor-I (IGF-I). It had been also proved that IGF-I could stimulate cell proliferation and suppress apoptosis which may be linked to bladder cancer [26]. In addition, inflammatory mediators such as C-reactive protein and interleukin-6 may contribute to the obesity-related bladder cancer risk as suggested by positive relation of circulating levels of inflammatory markers to bladder cancer mortality [27, 28]. However, further study into carcinogenic mechanisms between body weight and bladder cancer risk are needed.

The present meta-analysis has several key strengths. The present meta-analysis was only based on well-established prospective cohorts which had minimized recall and selection biases. The included studies with large sample sizes and long term follow-up durations enhanced the statistical power to detect more stable associations and provide more reliable estimation. If a true association between overweight and bladder cancer risk existed, the follow-up durations in our analyses should have been long enough to detect such an association. Furthermore, we have examined the exact shape of the dose-response relationship between BMI and bladder cancer risk. Another strength is the robustness of the findings from the multiple subgroup analyses. The overall findings were consistent with the results of the subgroup analyses independent of gender, age, year of publication, sample size, assessment of BMI, geographic location, physical activity and family history of cancer.

Our study has some limitations when we interpreting the current results. First, although heterogeneity was observed across studies when we conducted meta-analysis, we were able to reveal the major sources of heterogeneity *via* subgroup analyses based on gender, age, year of publication, sample size, assessment of BMI, geographic location, physical activity and family history of cancer. Furthermore, our sensitivity test showed the findings were robust, and potential bias was not suggested by the Begg’s or the Egger’s test. Second, all the included studies in our analysis were prospective cohort studies. However, differences among follow-up duration, assessment of BMI and measurement of bladder cancer end point, may hinder an estimate of the true effects. Third, residual confounding factor is always a major concern in the epidemiology studies. The majority of included studies had adjusted with some lifestyle variables, but we could not exclude the possibility that other uncontrolled or unmeasured confounding factors play roles in the summary associations. An additional limitation is that the results of our meta-analysis have limited generalisation to other regions (e.g. Latin America and Africa), though the geographical regions covered in this meta-analysis included Asia Europe, the USA and some multi-international centers.

To sum up, our dose-response meta-analysis on the basis of prospective cohort studies indicated an increased bladder cancer risk of 3.1% for each 5 kg/m^2^ increment on BMI, especially when BMI was higher than 30kg/m^2^. Given the alarm rising rates of overweight and obesity worldwide, further studies are required to confirm these findings and elucidate the pathogenic mechanisms.

## MATERIALS AND METHODS

### Search strategy

Two authors independently and systematically searched Pubmed and Embase databases up to December 01, 2016. Research articles were selected using the following terms: ”body mass index”, or ”BMI ”, or ”obesity”, or ”adiposity”, or ”overweight”; “bladder cancer”, or ”urinary bladder neoplasms” or ”urothelial carcinoma”. The search was focused on human studies, without any other restriction. We also scrutinised the reference lists of reviews, meta-analyses, and selected research articles to identify additional relevant studies. Additionally, in view of the large number of bladder cancers arising in China, we have also searched the China National knowledge infrastructure (CNKI).

### Selection criteria

We included studies that met the following criteria:(a) An original article; (b) A prospective cohort study; (c) Determining BMI at baseline and then recording the incidence of bladder cancer during follow-up; (d) Risk estimate as hazard ratio (HR), risk ratio (RR) or odd ratio (OR) with corresponding 95% confidence interval (CI) for more than three categories of BMI or providing sufficient data to estimate these. (e) RR and corresponding 95% CI at least with adjustment for age. If the studies were reported from the same or overlapping cohort, only the most recent and informative one would be included. Discrepancies between two investigators were solved by discussion.

### Data extraction

Two investigators independently extracted data, which were cross-checked by another investigator. A standardized data-collection protocol for each study included was used with following information: the first author, the year of publication, gender, study area, duration of follow-up, sample size, number of cases, ascertainment of exposure, cut off of exposure, and adjustments for confounders. If one study reported several risk estimates, we used the one from the main multivariable model which included more adjusted confounders. If the study didn’t provide total person-years or total participants for each group, we estimated them using the method carried out by Aune [7]. In addition, the quality of the included study was assessed by the Newcastle-Ottawa Scale (NOS).

BMI categories were defined according to the World Health Organization (WHO): Normal (BMI 18.5~25 kg/m^2^), Overweight (BMI 25~30 kg /m^2^), and Obesity (BMI ~30 kg/m^2^) [6].

### Statistical analysis

Meta-analyses were conducted to explore the relative risk of bladder cancer on overweight and obesity compared with normal BMI level. For studies that reported RR for several categories of BMI that fell into the category representing overweight or obesity, we pooled the RR weight by the inverse of their variance. Furthermore, meta-analysis with the assumptions of a random-effects model was conducted to calculate The summary relative risk (SRR), which incorporates between-study variability into the calculation [29]. A two-tail *p* value < 0.1 was considered statistically significant. Heterogeneity among studies was assessed using the *Q* or *I*^2^ statistics, which tested total variation across studies that was attributable to heterogeneity rather than to chance [30].

A dose-response analysis was conducted based on the category data of BMI, number of cases, person-years and logarithm of RRs and its corresponding standard error. The eligible studies should provide sufficient information across at least three categories of exposure. Among the studies, we assigned a median of BMI for each category. For the open-ended upper category, the amplitude was assumed the same as the previous one. We transformed category-specific risk estimates into estimates of the relative risk (RR) associated with every 5 *kg/m^2^* increase on BMI by used of the method of generalized least-squares for trend estimates.^9^ The potential nonlinear dose-response relationship between BMI and bladder cancer risk was examined using a two-stage hierarchical regression model [31]. Data were modeled with random-effects restricted to cubic spline models with 4 knots and using the Greenland and Longnecker method to estimate the covariances of multivariable-adjusted relative risks [32].

Sources of heterogeneity were explored by subgroup analyses based on gender, age, year of publication, sample size, assessment of BMI, geographic location, physical activity and family history of cancer. Sensitivity analyses were conducted to clarify whether the results were affected due to one single study by repeating the meta-analysis after omitting one study at a time. We inspected the funnel plots for asymmetry and with Egger’s test and Begg’s test to test publication bias. All statistical analyses were performed with STATA Statistical Software, version 12.0.
